# Physical Grinding of Prefabricated Co_3_O_4_ and MCM-22 Zeolite for Fischer–Tropsch Synthesis: Impact of Pretreatment Procedure on the Dispersion and Catalytic Performance

**DOI:** 10.3390/molecules29061283

**Published:** 2024-03-14

**Authors:** Hua-Ping Ren, Zhi-Xia Xie, Shao-Peng Tian, Si-Yi Ding, Qiang Ma, Yu-Zhen Zhao, Zhe Zhang, Jiao-Jiao Fu, Qing-Qing Hao

**Affiliations:** 1 Technological Institute of Materials & Energy Science (TIMES), Xi’an Key Laboratory of Advanced Photo-Electronics Materials and Energy Conversion Device, School of Electronic Information, Xijing University, Xi’an 710123, China; renhuaping@xijing.edu.cn (H.-P.R.); tianshaopeng@xijing.edu.cn (S.-P.T.); dingsiyi@xijing.edu.cn (S.-Y.D.); maqiang@xijing.edu.cn (Q.M.); zhaoyuzhen@xijing.edu.cn (Y.-Z.Z.); zhangzhe@xijing.edu.cn (Z.Z.); fujiaojiao@xijing.edu.cn (J.-J.F.); 2 School of Chemical Engineering, Northwest University, Xi’an 710069, China; xiezx@nwu.edu.cn

**Keywords:** Fischer–Tropsch, MCM-22, cobalt, physical grinding, dispersion

## Abstract

To improve the mess-specific activity of Co supported on zeolite catalysts in Fischer–Tropsch (FT) synthesis, the Co-MCM-22 catalyst was prepared by simply grinding the MCM-22 with nanosized Co_3_O_4_ prefabricated by the thermal decomposition of the Co(II)-glycine complex. It is found that this novel strategy is effective for improving the mess-specific activity of Co catalysts in FT synthesis compared to the impregnation method. Moreover, the ion exchange and calcination sequence of MCM-22 has a significant influence on the dispersion, particle size distribution, and reduction degree of Co. The Co-MCM-22 prepared by the physical grinding of prefabricated Co_3_O_4_ and H^+^-type MCM-22 without a further calcination process exhibits a moderate interaction between Co_3_O_4_ and MCM-22, which results in the higher reduction degree, higher dispersion, and higher mess-specific activity of Co. Thus, the newly developed method is more controllable and promising for the synthesis of metal-supported catalysts.

## 1. Introduction

Fischer–Tropsch (FT) synthesis is an efficient process for converting syngas (CO + H_2_) to super clean fuels and high-value-added fine chemicals [1,2,3]. However, the product distribution of FT synthesis is very broad because of the Anderson–Schulz–Flory (ASF) polymerization mechanism [4,5]. Thus, controlling the product distribution is crucial for the direct synthesis of target products (diesel, gasoline, et al.) with high selectivity through the FT process [6,7]. Cobalt has been widely used as an effective catalyst for FT synthesis due to its high activity, high resistance to deactivation, low water–gas shift activity, and lower price compared with noble metals [1,5]. Consequently, bifunctional catalysts, that is, combinations of cobalt with various solid acids for cracking and isomerizing the FT hydrocarbons, have been extensively studied to circumvent the ASF distribution and produce gasoline or diesel-range hydrocarbons with high selectivity [4,8,9].

Cobalt supported on zeolites has proven to be very effective, and the traditional FT product distribution can be significantly changed with much-increased selectivity to liquid fuels [10,11,12]. A cobalt nitrate precursor is often used owing to its high solubility, which allows for high metal loading in a single impregnation step. However, due to the infiltration of cobalt species into the micropores of zeolite and the strong interaction between Co and zeolite, a poor reduction degree of cobalt is frequently obtained, which results in lower mass-specific activity [13,14,15,16,17]. Moreover, inhomogeneous size distributions of cobalt are frequently obtained because the Co is simultaneously located on the internal surface and external surface of the microporous channel, which results in a higher deactivation rate [18,19,20]. Thus, developing a novel method for the preparation of an efficient Co/zeolite catalyst with highly dispersed and reducible Co is crucial for the development of a bifunctional FT catalyst with higher mass-specific activity of Co.

In essence, bifunctional Co-zeolite-based catalysts are combinations of FT-active metal Co with zeolite for cracking and isomerizing the FT hydrocarbons. In fact, except for the direct deposition of Co on or in zeolite, the physical mixing of FT catalysts (Co/SiO_2_, Co/Al_2_O_3_) and zeolite has been extensively studied [21,22,23]. However, the distance between the two kinds of active sites, i.e., FT-active sites (Co/SiO_2_, Co/Al_2_O_3_) and acid sites, in zeolite is relatively far, which results in a weaker synergistic effect. Therefore, the physical mixing of prefabricated nanosized Co_3_O_4_ and zeolite is a prospective strategy for obtaining efficient bifunctional FT catalysts with higher mass-specific activity. 

As reported in the literature [24,25,26,27,28,29,30,31,32,33], the chelate-assisted method has been widely used to improve the dispersion of metal for supported catalysts. In our previous work, Co/SiO_2_ catalysts with nanosized and homogenous size distributions of Co can be prepared by the thermal decomposition of Co(II)-glycine complexes prepared by the reaction of glycine with cobalt hydroxide [34]. Based on this work, nanosized Co_3_O_4_ should be prefabricated by the thermal decomposition of Co(II)-glycine complexes in the absence of support. Consequently, the interaction between Co and zeolite over the physical mixing of prefabricated nanosized Co_3_O_4_ and zeolite may be decreased in comparison with Co/zeolite prepared by the incipient impregnation method. MCM-22 zeolite with MWW topology is one of the most investigated 2D zeolites due to its modifiable arrangement of thin layers (ca. 2.5 nm thick) and unique pore systems, which consist of two different pores [35,36], i.e., intralayer 10-member ring (MR) sinusoidal channels and interlayer 12MR supercages interconnected through 10MR windows. It has been reported that Co/MCM-22 is a promising catalyst for FT synthesis with narrowed product distributions [17,37]. 

In this work, using a newly developed method, the Co_3_O_4_ was prepared by the thermal decomposition of Co(II)-glycine complexes prepared by the reaction of glycine with cobalt hydroxide. Then, the FT bifunctional catalyst was obtained by the direct physical mixing of prefabricated nanosized Co_3_O_4_ and MCM-22 zeolite. Moreover, the impacts of the template removal order of MCM-22 on its structure, acidity, and particle size of Co_3_O_4_ were comparatively investigated. For comparison, the Co-supported catalyst was also prepared by co-impregnation with the impregnation solution containing cobalt nitrate. Significantly, the Co-MCM-22 catalyst prepared by the physical mixing of prefabricated nanosized Co_3_O_4_ and MCM-22 exhibited smaller Co particle size and narrower size distribution, which resulted in higher activity and stability in the FT reaction.

## 2. Results and Discussion

### 2.1. The Effect of Ion Exchange and Calcination Sequence on the Structural and Acidic Properties of MCM-22

Generally, the as-synthesized zeolites have a structure-directing agent (SDA) in their framework. Thus, to convert the as-synthesized sample to H-type zeolite with acid sites, two-step calcination is needed. Typically, the first step of calcination is to remove the SDA and convert the as-synthesized sample to Na-type zeolite. Then, the Na-type zeolite is ion-exchanged with NH_4_^+^ to obtain the NH_4_^+^-type zeolite. Finally, after the second step of calcination, the H-type zeolite can be obtained. In this work, we attempt to eliminate the first calcination to remove the SDA, that is, the as-synthesized MCM-22 (including SDA) directly ion-exchanged with NH_4_^+^ to obtain the NH_4_^+^-type MCM-22 (including SDA). Then, removing SDA and converting NH_4_^+^ to H^+^ was achieved through only a one-step calcination procedure. As mentioned in the introduction, the infiltration of cobalt species into the micropores of zeolite will result in a strong interaction between Co and zeolite. Thus, it is expected that the interaction between Co species and NH_4_^+^-type MCM-22 without removing the SDA could be decreased because of the existence of HMI in the micropores of MCM-22. However, whether the ion exchange and calcination sequence have an effect on the physical and chemical properties of MCM-22 zeolites is unknown. Thus, the influence of the ion exchange and calcination sequence on the structure and acidic properties of MCM-22 was investigated first. 

**Figure 1:**
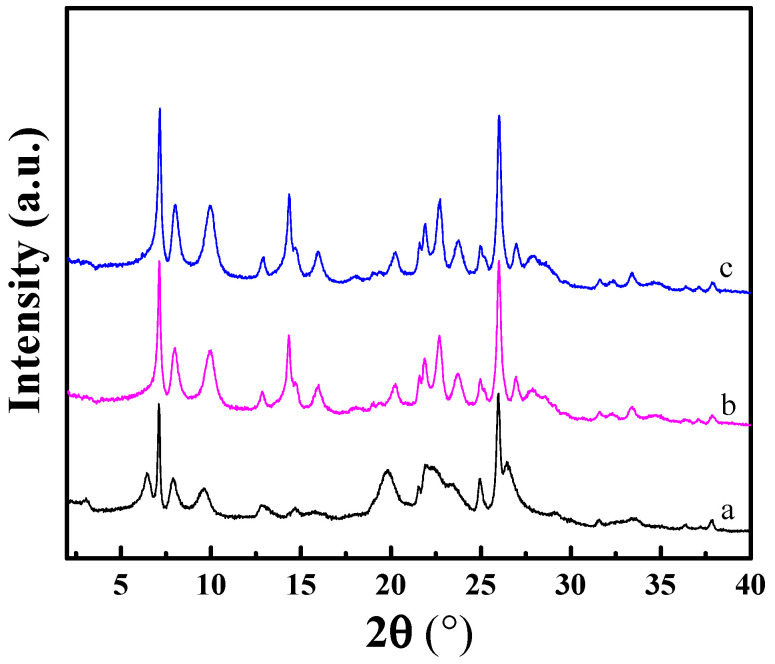
XRD patterns of the MCM-22 (P) (**a**), H-MCM-22(1) (**b**), and H-MCM-22(2) (**c**).

The XRD patterns of the MCM-22(P) and H-MCM-22 samples prepared by different ion exchange sequences are shown in Figure 1. The XRD pattern of MCM-22(P) shows clear (001) and (002) diffraction peaks at 2 theta of 3.2 and 6.5° corresponding to d-spacings of 2.70 and 1.35 nm, indicating the ordered layered structure of MCM-22(P) with the vertically aligned layers along the *c*-axis [16,34]. Before template removal, the diffraction peaks in the 2 theta range of 12–30° are broad and some of them overlap. Significantly, the diffraction peaks of two H-MCM-22 prepared by different ion exchange sequences are very similar, indicating that the ion exchange and calcination sequence method has less effect on the crystal structure of H-MCM-22. 

**Figure 2:**
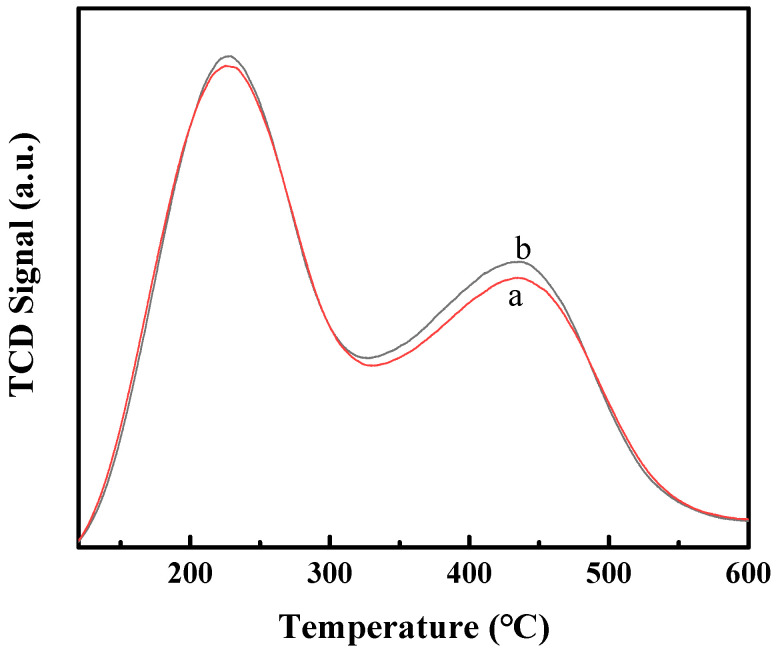
NH_3_-TPD profiles of H-MCM-22(1) (**a**) and H-MCM-22(2) (**b**).

Commonly, the acidic properties of zeolites can be characterized by using the NH_3_-TPD technique to estimate the amount and distribution of weak and strong acid sites and using traditional pyridine-IR or novel Diffuse Reflectance Infrared Fourier Transform Spectroscopy (DRIFTS) without molar extinction coefficients [38] to estimate the Lewis and Brønsted sites. In this work, the acidic properties of H-MCM-22 prepared by different ion exchange sequences are estimated by the NH_3_-TPD technique. As shown in Figure 2, two clear NH_3_ desorption peaks can be seen for both H-MCM-22, which correspond to the weak and strong acidic sites. Significantly, the NH_3_-TPD profiles of the two samples are almost identical with each other. This observation indicates that the ion exchange sequence and the calcination method have less effect on the acidic properties of H-MCM-22. These results may be related to the unique SDA of hexamethyleneimine (HMI). The presence of HMI in the framework of MCM-22 does not have a steric hindrance effect on the ion exchange of NH_4_^+^ to Na^+^ due to the smaller molecular size of HMI. Moreover, the thermal stability of MCM-22 was indirectly proved by the slight change in structure and acidity after calcining twice. Therefore, for the preparation of H^+^-type MCM-22 zeolite, the first calcination procedure for removing the SDA can be eliminated, which can lower the cost of the synthesis of H-MCM-22 zeolite. 

### 2.2. The Crystal Structure of Co_3_O_4_ and Co-MCM-22 Catalyst

**Figure 3:**
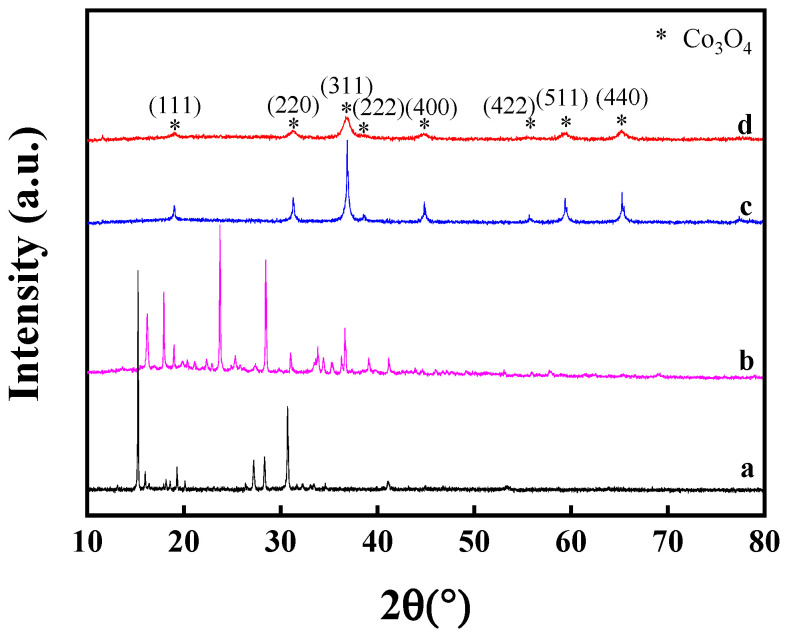
XRD patterns of the Co(NO_3_)_2_·6H_2_O (**a**), Co(II)-glycine complex (**b**), Co_3_O_4_ prepared using Co(NO_3_)_2_·6H_2_O as precursor (**c**), and Co_3_O_4_ prepared using Co(II)-glycine as precursor (**d**).

As described in Section 3, the Co(II)-glycine complex is prepared by the reaction of glycine and Co(OH)_2_. As shown in Figure 3, the diffraction peaks assigned to Co(OH)_2_ species cannot be seen over the Co(II)-glycine complex, indicating that the Co(OH)_2_ is fully coordinated by the glycine and giving the homogenous Co complex with stable and definite structure, namely, Co(glycine)_2_(H_2_O)_2_. After the calcination of the Co(II)-glycine complex and Co(NO_3_)_2_·6H_2_O at 400 °C, the diffraction peaks of cobalt species are assigned to Co_3_O_4_ without other crystalline phases. Significantly, the peak width at half the height of Co_3_O_4_ prepared by the thermal decomposition of the Co complex is bigger than that of Co_3_O_4_ prepared by the thermal decomposition of Co(NO_3_)_2_·6H_2_O. This result indicated that the particle size of Co_3_O_4_ derived from the Co(II)-glycine complex is smaller than that of Co_3_O_4_ derived from Co(NO_3_)_2_·6H_2_O. Therefore, the thermal decomposition of the Co(II)-glycine complex is an effective method for the preparation of nanosized and homogeneous Co_3_O_4_ crystals.

**Figure 4:**
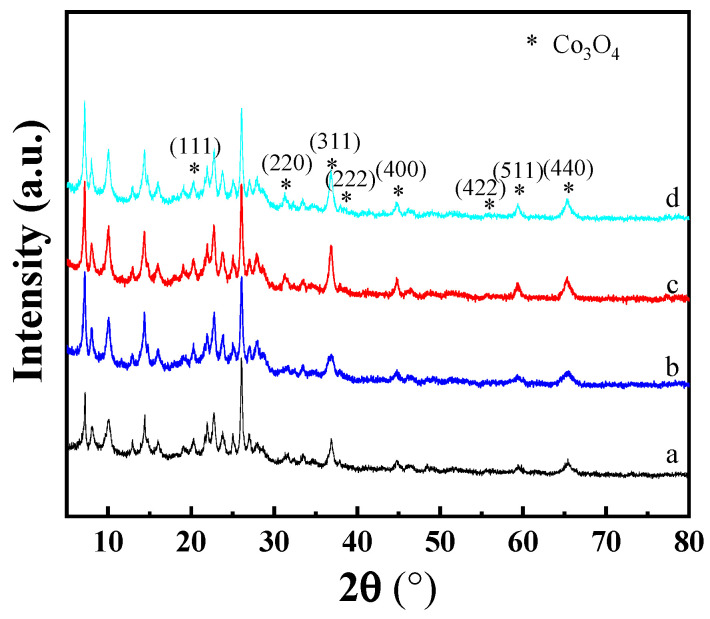
XRD patterns of the Co/MCM-22 (**a**), Co-MCM-22(1) (**b**), Co-MCM-22(2) (**c**), and Co-MCM-22(1)-500 (**d**).

The Co-based catalysts were prepared by three different methods, i.e., Co/MCM-22 prepared by the incipient impregnation method, Co-MCM-22(1) prepared by the physical mixing of Co_3_O_4_ and H-MCM-22, and Co-MCM-22(2) prepared by the physical mixing of Co_3_O_4_ and NH_4_^+^-MCM-22 (including HMI in the micropores) followed by calcination at 550 °C to remove HMI and obtain the H-MCM-22. As shown in Figure 4, the preparation methods have influenced the particle size of Co_3_O_4_ over MCM-22 zeolite. Significantly, the Co-MCM-22(1) shows the smallest particle size of Co_3_O_4_, in which the Co_3_O_4_ particle size is similar to that of prefabricated Co_3_O_4_, indicating that the grinding process has a limited effect on the particle size of Co_3_O_4_. It should be noted that the diffraction peak intensity of MCM-22 in the Co/MCM-22 prepared by the impregnation method is lower than that of Co_3_O_4_-MCM-22. This observation can be reasonably attributed to the entrance of Co_3_O_4_ into the micropores of MCM-22, which results in crystal imperfection and a decrease in peak intensity. Because the Co_3_O_4_ is located on the internal and external micropores of MCM-22 for Co/MCM-22 simultaneously, the Co_3_O_4_ particle size estimated from the XRD is an average value, which is slightly larger than that of the Co-MCM-22(1). For the Co-MCM-22(2) prepared using a tentative strategy, the particle size of Co_3_O_4_ is larger than those of Co/MCM-22 and Co-MCM-22(1). Thus, the expected result was not achieved. The larger particle size of Co_3_O_4_ over Co-MCM-22(2) can be attributed to the calcination procedure for removing the SDA of MCM-22, which results in the sintering of Co_3_O_4_ at high temperatures of 550 °C [1,19]. This explanation can be certified by the increased Co_3_O_4_ particle size of Co-MCM-22(1)-500 prepared by treating the Co-MCM-22(1) at 550 °C for 2 h.

### 2.3. Reduction Behavior and the Dispersion of Co Catalysts

**Figure 5:**
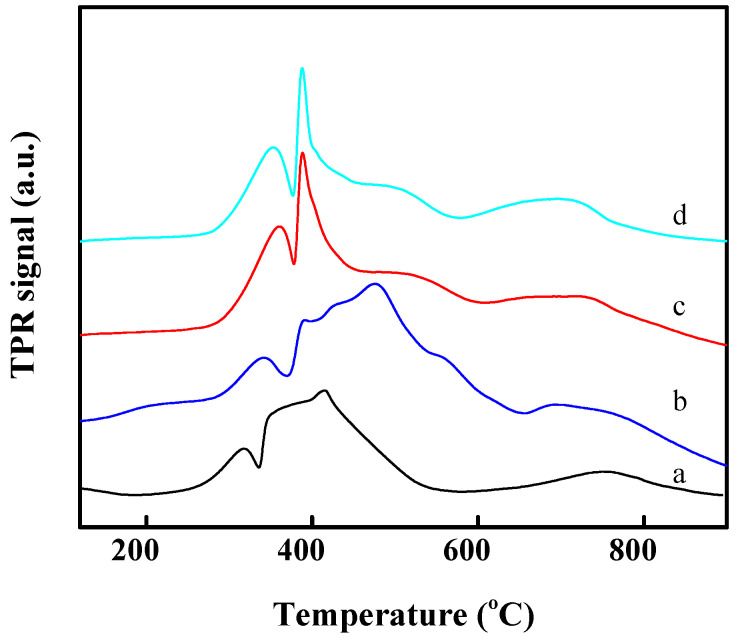
H_2_-TPR profiles of the Co/MCM-22 (**a**), Co-MCM-22(1) (**b**), Co-MCM-22(2) (**c**), and Co-MCM-22(1)-500 (**d**).

Our H_2_-TPR measurement was used to evaluate the reduction behavior of the catalysts (Figure 5). The Co supported on MCM-22 catalysts shows two discrete peaks in the range of 250–650 °C, which are assigned to the two-step reduction of Co_3_O_4_ to CoO and CoO to metallic Co, respectively. As shown in Figure 5, the temperature for the reduction of Co_3_O_4_ to CoO (the first reduction peak) over Co/MCM-22 is clearly lower than those of Co_3_O_4_-MCM-22 prepared by the physical mixing method, indicating the existence of external Co_3_O_4_ with a bigger particle size over Co/MCM-22 [17]. Significantly, the reduction peak of CoO to metallic Co over Co-MCM-22(1) is in the broader range of 350–600 °C, which can be attributed to the smaller and moderate interaction between the Co species and MCM-22 zeolite. In contrast, the sharp reduction peak at about 400 °C over Co-MCM-22(2) and Co-MCM-22(1)-500 can be reasonably attributed to the bigger Co_3_O_4_ particle size induced by the higher temperature treating procedure. 

**Figure 6:**
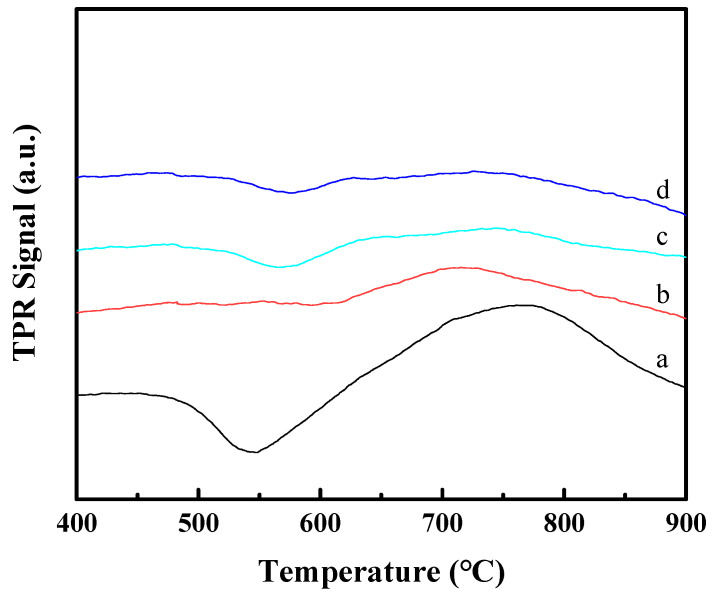
H_2_-TPR profiles of the Co/MCM-22 (**a**), Co-MCM-22(1) (**b**), Co-MCM-22(2) (**c**), and Co-MCM-22(1)-500 (**d**) after reduction at 400 °C for 4 h.

It should be noted that there are clear reduction peaks in the range of 650-850 °C, which are attributed to the reduction in Co_2_SiO_4_-like species [16,17]. However, it must be pointed out that the Co_2_SiO_4_-like species can be formed in the temperature-programmed process above 400 °C for the H_2_-TPR measurement, while the reduction process before the FT reaction used in this work is at 400 °C for 4 h. Therefore, to demonstrate the formation reasons and estimate the reduction degree of different catalysts, the H_2_-TPR measurements were tested after the in situ reduction at 400 °C for 4 h. As shown in Figure 6, the Co/MCM-22 exhibits a clear reduction peak in the range of 650–850 °C, while the reduction peak in this temperature range for those three Co_3_O_4_-MCM-22 catalysts is not very obvious. These observations indicate that the irreducible Co_2_SiO_4_-like species at 400 °C can be avoided to a certain extent by using the physical mixing method to prevent Co species from moving into the micropores of MCM-22 zeolite. Moreover, the reduction degrees of the four catalysts are summarized in Table 1. The reduction degree of Co_3_O_4_-MCM-22 is clearly higher than that of Co/MCM-22. 

Commonly, the surface Co^0^ density (Co dispersion) of the catalyst is determined by the particle size and the reduction degree of Co. H_2_-chemisorption is an effective technique to obtain the actual surface Co^0^ density and the dispersion (normalized by the total moles of Co over the catalyst). As shown in Table 1, the Co/MCM-22 exhibits the lowest Co dispersion (about 3.0%) in the four catalysts in this work, which can be attributed to the lower reduction degree and large particle size of Co. Moreover, the Co-MCM-22(1) shows the highest Co dispersion in the three catalysts prepared by the physical mixing method. This result can be attributed to the moderate interaction between Co and MCM-22, which results in a higher reduction degree and smaller Co particle size.

### 2.4. FT Performance

**Figure 7:**
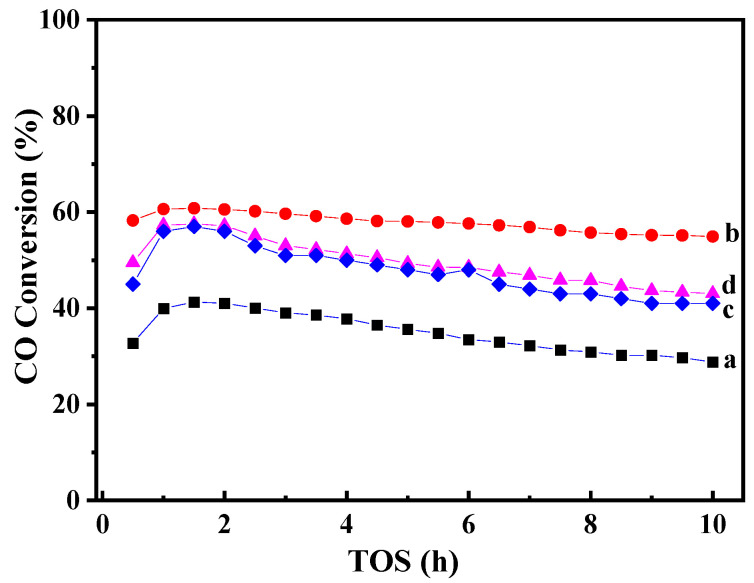
Time-on-stream CO conversion over Co/MCM-22 (**a**), Co-MCM-22(1) (**b**), Co-MCM-22(2) (**c**), and Co-MCM-22(1)-500 (**d**).

The catalyst was evaluated for FT synthesis under the conditions of 250 °C, 1 MPa, H_2_/CO = 2, and W/F = 5.0 g h mol^−1^. As shown in Figure 7, the CO conversions at a steady state (at TOS = 10 h) over the catalysts are increased in the order of Co-MCM-22(1) > Co-MCM-22(1)-500 = Co-MCM-22(2) > Co/MCM-22, which is consistent with the changing trend of Co^0^ dispersion in Table 1. Moreover, the catalytic stability of Co-MCM-22(1) within the reaction time is obviously higher than that of others. In contrast, the deactivation rate of Co/MCM-22 is clearly higher than those of Co-MCM-22 catalysts prepared by the physical mixing method. Based on the theoretical calculation, the Ostwald ripening rate could be suppressed further by preparing the homogeneously distributed metal particles with identical sizes. [19] Therefore, the higher stability of Co-MCM-22(1) can be reasonably explained by the homogeneous size distribution of Co particles and moderate interaction between Co and MCM-22 zeolites. In addition, the Co/MCM-22 is prepared by the incipient impregnation method, which makes a major part of the Co species infiltrate into the micropores of zeolite. The coke deposition will be formed over acid sites in the micropores due to the second cracking reaction of FT products, which will result in the coverage of Co^0^ active sites and the deactivation of the Co/MCM-22 [11,12,39].

**Figure 8:**
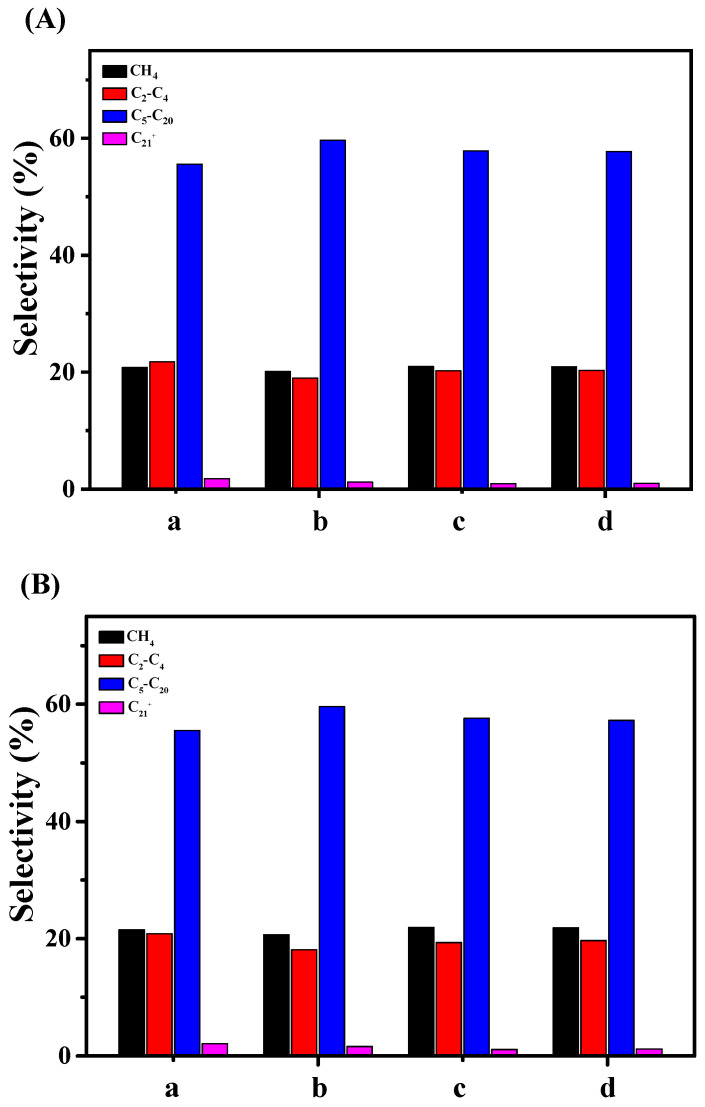
The carbon number distribution of FT hydrocarbons at TOS of 5 h (**A**) and 10 h (**B**) over Co/MCM-22 (a), Co-MCM-22(1) (b), Co-MCM-22(2) (c), and Co-MCM-22(1)-500 (d).

Figure 8 shows the product distribution of the FT synthesis at TOS of 5 h and 10 h over these catalysts. The product distribution at TOS of 5 h and 10 h are very similar due to the slight change in CO conversion in this period. The selectivity of CH_4_ and C_2_-C_4_ over the four catalysts is higher than in the previous report [16] due to the higher reaction temperature in this work. However, the product distribution over the four catalysts in this work is very similar. It must be pointed out that the particle size of Co^0^ in the range of 10–30 nm exhibits a slight influence on the product distribution [13,14,40]. As shown in Table 1, the particle size of Co^0^ in this work is in the range of 14–21 nm, which results in the selectivity of the FT primary product being very similar. The selectivity of C_21_+ is obviously restrained due to the second cracking reaction of the FT product over the acidic sites of MCM-22, while the selectivity of C_5_–C_20_ is significantly increased compared with that of Co/SiO_2_ catalysts without acidic sites. It should be noted that the selectivity of C_21_+ over Co-MCM-22(1) is slightly higher than that of the other three catalysts. This result can be reasonably attributed to the higher CO conversion and the not-covered acidic site in the micropores of MCM-22. 

## 3. Materials and Methods

### 3.1. Materials

Hexamethyleneimine (HMI, 99%) and colloidal silica (Ludox, AS-40, 40%) were purchased from Sigma-Aldrich, Shanghai, China. Co(NO_3_)_2_·6H_2_O (98.5%), glycine (99%), sodium hydroxide (NaOH, 96%), sodium, aluminate (NaAlO_2_, 41% Al_2_O_3_), and ammonium nitrate (NH_4_NO_3_, 99%) were provided by Sinopharm Chemical Reagent Co., Ltd., Shanghai, China. All reagents were used directly without further treatment.

### 3.2. Preparation of Nanosized Co_3_O_4_

The nanosized Co_3_O_4_ was prepared by the thermal decomposition of Co(II)-glycine complexes. Typically, the Co(II)-glycine precursor was prepared by the reaction of glycine with Co(OH)_2_ (mole ratio of glycine/Co(OH)_2_ = 3). The Co(OH)_2_ was prepared by the reaction of Co(NO_3_)_2_ with NaOH using the molar ratio of OH^−^/Co^2+^ = 2. After the formation of Co(OH)_2_ precipitate, the Co(OH)_2_ was recovered by filtration and washing with deionized water thoroughly, and then the wet Co(OH)_2_ was dried at 80 °C for 12 h. For the preparation of the Co(II)-glycine complex, the Co(OH)_2_ powder was slowly added to the 0.2 mol/L glycine aqueous solution (in which the molar ratio of glycine to Co(OH)_2_ is 3) at 80 °C. After the addition of Co(OH)_2_ powder, the solution was still stirred for 2 h at 80 °C, and then most of the water was removed through rotary evaporation. Finally, the Co(II)-glycine complex was obtained after drying the samples overnight at 90 °C. 

### 3.3. Preparation of MCM-22 Zeolite

MCM-22(P) was synthesized based on the reported method in the literature [35]. Typically, 0.15 g of NaAlO_2_ and 0.18 g of NaOH were dissolved in 25.2 mL of deionized water, and then 4.7 g of colloidal silica was added. The mixture was stirred for 0.5 h, and then 1.1 g of HMI was added. The final molar composition of the mixture was 0.07 Na_2_O/1 SiO_2_/0.02 Al_2_O_3_/0.35 HMI/ 45 H_2_O. The reaction mixture was transferred into 50 mL Teflon-lined stainless-steel autoclaves. The autoclaves were tumbled at 60 rpm in an oven at 150 °C. After 7 days, the autoclaves were fast-cooled in water, and the products were centrifuged and washed with deionized water until the pH = 8. MCM-22(P) was obtained after drying the samples overnight at 90 °C. A part of the MCM-22(P) was calcined at 550 °C for 6 h to obtain the Na-type MCM-22 zeolite. Consequently, the Na-type MCM-22 was ion-exchanged 3 times in a 1 mol/L NH_4_NO_3_ solution at 80 °C for 2 h. Finally, the NH_4_^+^-type MCM-22 was calcined at 500 °C for 2 h to obtain the H-type MCM-22 (H-MCM-22(1)). Another part of MCM-22(P) was directly ion-exchanged 3 times in a 1 mol/L NH_4_NO_3_ solution at 80 °C for 2 h to obtain the NH_4_^+^-type MCM-22 without removing the structure direct agent (HMI). Finally, the NH_4_^+^-type MCM-22 was calcined at 500 °C for 4 h to obtain the H-type MCM-22 (H-MCM-22(2)). 

### 3.4. Preparation of Co-Based Catalyst

The Co-MCM-22 catalyst was prepared by the direct physical mixing of prefabricated nanosized Co_3_O_4_ and MCM-22. Typically, the nanosized Co_3_O_4_ and H-MCM-22 were mixed in an agate mortar for 10 min, which is denoted as Co-MCM-22(1). The nanosized Co_3_O_4_ and NH_4_^+^-type MCM-22 were mixed in an agate mortar for 10 min, and then the catalyst was calcined at 500 °C for 4 h to remove the structure direct agent (HMI) and convert the NH_4_^+^ to H^+^ of MCM-22, which is denoted as Co-MCM-22(2). The metallic Co loading for all the catalysts was 10 wt.%.

For comparison, the Co/MCM-22 was prepared by the incipient impregnation method. The cobalt nitrate (Co(NO_3_)_2_·6H_2_O, 99.0%) was used as the cobalt precursor. The catalysts were dried at 120 °C for 12 h, and then calcined at 200 °C for 2 h in air by programmed heating with a rate of 2 °C min^−1^. 

### 3.5. Characterizations

The NH_3_-TPD measurements were performed with a BELCAT II (Microtrac BEL, Osaka, Japan) instrument. Typically, 0.05 g of the sample was preheated with flowing He at 550 °C for 1 h and then cooled to 120 °C. Subsequently, the sample was exposed to an NH_3_-He mixture (5 vol% NH_3_) for 0.5 h. After this, the system was purged for 1 h under a flow of He at the same temperature. After this, NH_3_-TPD was performed by raising the temperature to 600 °C at a heating rate of 10 °C/min under a He flow of 30 cm^3^/min. XRD measurement was performed on an X-ray diffractometer (D8 Advance) with Cu Kα radiation operated at 40 kV and 40 mA. The speed of scanning is 4°/min with a step size of 0.02°. The average crystallite size of Co_3_O_4_ over the catalysts was estimated using (311) diffraction lines (2θ at about 37.0°) according to Scherrer’s equation. The H_2_-TPR was carried out on a BELCAT II (MicrotracBEL) instrument. Notably, 0.05 g of the catalysts was first purged in a flow of argon at 200 °C for 30 min. After the temperature decreased to 35 °C, the catalysts were heated to 900 °C at a heating rate of 10 °C/min under 10 vol.% hydrogen–argon mixtures with a flow rate of 30 cm^3^/min. The reduction degree of cobalt was determined by the O_2_ pulse titration method. Firstly, about 0.1 g of catalyst was reduced in situ for 6 h at 500 °C using pure hydrogen. Afterwards, the temperature of the sample was decreased to 400 °C, and it was flushed with pure Ar for 1 h. At the same temperature, 3 vol. % O_2_ was injected with a pulse mode to oxidize the reduced catalyst. The reduction degree (RD, %) of the catalyst was estimated based on the consumption of oxygen assuming that metallic Co was converted to Co_3_O_4_. H_2_-chemisorption measurements were performed on a Micromeretics ASAP 2020C instrument to evaluate the Co dispersion. Before measurement, the sample was reduced on the analysis station in situ in flowing H_2_ at 500 °C for 6 h. Afterwards, the temperature was decreased to 100 °C and the H_2_-chemisorption was measured at this temperature. The H_2_ uptakes and Co dispersion (D, %) were determined using the method reported in the literature. Assuming the hemispherical geometry of the metallic Co, with a surface atomic density of 14.6 atoms/nm^2^, the particle sizes of Co^0^ were calculated using the *d*(Co)_H_ = 96 × RD/D formula. 

### 3.6. Catalytic Reactions

The catalytic performance of the catalysts in FT synthesis was tested in a fixed-bed reactor. Typically, 0.5 g of catalyst (40–60 mesh diluted with quartz sands) was firstly reduced in situ in a flow of pure H_2_ (50 cm^3^/min) at 400 °C for 6 h. And then, the temperature was decreased to 190 °C and the syngas (H_2_/CO = 2, 4% Ar as an internal standard) was fed into the reactor. The reaction conditions are at 250 °C, 1.0 MPa, and W/F = 5.0 g·h·mol^−1^. To prevent condensation of the products, the pipeline from the outlet of the reactor to the inlet of the gas chromatography (GC) was heated at 180 °C. The hydrocarbons in the effluent were online and were analyzed by a GC with an HP-PONA capillary column (0.20 mm × 50 m, 0.5 μm) and a flame ionization detector (FID) (SP-3420A, Beifen-Ruili Analytical Instrument (Group) Co., Ltd., Beijing, China). The CO, CH_4_, Ar, and CO_2_ in the effluent were online and were analyzed by a GC with a packed activated carbon column and a TCD detector (SP-3420A). The selectivity for hydrocarbons was calculated based on carbon number.

## 4. Conclusions

In summary, nanosized Co_3_O_4_ was prefabricated by the thermal decomposition of the Co(II)-glycine complex prepared by the reaction of glycine and Co(OH)_2_. MCM-22-supported Co catalysts were prepared by the physical mixing of prefabricated Co_3_O_4_ with H-MCM-22 and NH_4_^+^-MCM-22. This is an effective strategy for improving the dispersion of Co and the mess-specific activity of Co catalysts in FT synthesis compared to the impregnation method. It is found that the dispersion, particle size distribution, and reduction degree of Co were significantly influenced by the ion exchange sequence and calcination conditions, although the ion exchange sequence and calcination conditions have a limited impact on the structure and acidic properties of MCM-22 zeolite. In comparison to the Co/MCM-22 prepared by the traditional impregnation method, the Co-MCM-22(1) prepared by the newly developed method, i.e., simply mixing the nanosized Co_3_O_4_ and H-MCM-22, exhibited higher mess-specific activity of Co. Thus, the newly developed method is more controllable and promising for the synthesis of Co-based catalysts for FT synthesis.

## Figures and Tables

**Table 1:** Crystal sizes and reduction degree of cobalt over different catalysts.

Catalysts	Co Size (nm)		Reduction Degree ^b^ (RD, %)	Co Dispersion ^c^ (D, %)
	d(Co_3_O_4_) ^a^	d(Co)_H_^d^		
Co_3_O_4_(G)	12.1	---	---	---
Co_3_O_4_(N)	34.5	---	---	---
Co/MCM-22	20.0	21.4	67.0	3.0
Co-MCM-22(1)	13.4	14.9	82.1	5.3
Co-MCM-22(2)	18.5	20.4	87.3	4.1
Co-MCM-22(1)-500	19.5	19.7	86.2	4.2

^a^ Estimation derived from Scherrer’s equation using (311) diffraction lines (2θ at about 37.0°). ^b^ The reduction degree (RD, %) was estimated based on the consumption of oxygen. ^c^ Co dispersion (D, %) was calculated using H_2_ uptakes in H_2_-chemisorption. ^d^ The particle sizes of Co^0^ were calculated using d(Co)_H_ = 96 × RD/D formula.

## Data Availability

The data presented in this study are included in the article.
